# Diabetes and metabolic syndrome in adults with malaria and associations with severe disease: results from two tertiary hospitals in Cameroon

**DOI:** 10.1186/s12879-025-11389-1

**Published:** 2025-08-22

**Authors:** George Awungafac, Sylvain Raoul Simeni Njonnou, Michel Noubom, Leila Ingrid Angeline Avezo’o Libon, Caroline Ngunyi Gesu, Anna Färnert, Siméon Pierre Choukem, Katja Wyss

**Affiliations:** 1 Department of Medicine Solna, Karolinska Institutet, Stockholm, Sweden; 2 Department of Internal Medicine and Specialties, Faculty of Medicine and Pharmaceutical Science, University of Dschang, Dschang, Cameroon; 3 Internal Medicine Unit, Dschang Regional Hospital Annex, Dschang, Cameroon; 4 Department of Microbiology, Haematology and Immunologyof the, Faculty of Medicine and Pharmaceutical Sciences of Dschang, Dschang, Cameroon; 5 Laboratory Unit, Dschang Regional Hospital Annex, Dschang, Cameroon; 6 Department of Clinical Sciences, Faculty of Health Sciences, University of Buea, Buea, Cameroon; 7 Department of Medical Laboratory Sciences, Faculty of Health Sciences, University of Buea, Buea, Cameroon; 8 Blood Bank Unit, Bafoussam Regional Hospital, Bafoussam, Cameroon; 9 Department of Infectious Diseases, Karolinska University Hospital, Stockholm, Sweden; 10 Health and Human Development Research Network, Douala, Cameroon

**Keywords:** Severe malaria, Diabetes, Metabolic syndrome, Obesity

## Abstract

**Background:**

The prevalence of lifestyle-associated diseases is increasing in sub-Saharan Africa. This study investigated the prevalence of metabolic co-morbidities in adults with malaria and whether type 2 diabetes, obesity, and metabolic syndrome (Met-S) affect the severity of malaria in adults living in a malaria-endemic setting.

**Methods:**

We conducted a cross-sectional study with prospective inclusion of patients at two tertiary-level public hospitals in Bafoussam and Dschang (West Region, Cameroon). Adults (≥ 21 years) diagnosed with malaria were included. Malaria severity was determined following the WHO criteria. All patients were assessed for diabetes, obesity, and Met-S, according to the International Diabetes Federation. Additional host factors investigated included age, sex, HIV infection, sickle cell trait and blood group. Multivariable logistic regression was used to assess potential associations with severe malaria.

**Results:**

Among 289 adults diagnosed with *P. falciparum* malaria, 120 (41.6%) fulfilled at least one criterion for severe malaria. Diabetes was found in 26/120 (21.7%) of patients with severe and 10/169 (6.0%) with non-severe malaria (*p* < 0.001). Met-S was diagnosed in 39/120 (32.5%) severe and 27/166 (16.3%) non-severe cases, respectively (*p* = 0.001). Obesity was similarly detected in severe (23/103; 22.3%) and non-severe cases (34/148; 23.0%). In multivariable analyses, diabetes (aOR = 3.24; 95%CI, 1.38—7.62) and Met-S (aOR = 3.18; 95%CI, 1.60—6.37) were independently associated with severe malaria.

**Conclusion:**

A high prevalence of diabetes and metabolic comorbidities was found among adults diagnosed with malaria in hospital settings in an endemic area. Diabetes and metabolic syndrome, but not obesity alone, were identified as risk factors for severe malaria. Investigation for diabetes should be considered in adults with severe malaria.

**Supplementary Information:**

The online version contains supplementary material available at 10.1186/s12879-025-11389-1.

## Background

Sub-Saharan Africa is experiencing a rapid increase in the number of people with type 2 diabetes [1, 2]. The International Diabetes Federation (IDF) estimated that 24 million people are living with diabetes in Africa, and more than half are unaware of their condition [3, 4]. At the same time, the region accounted for approximately 94% of the global malaria cases and 95% of deaths [5].

Diabetes and obesity are known to affect the disease course of several infections [6, 7], with more severe presentations in, for example, influenza [8, 9], Covid-19 [10] and streptococcal infections [11]. Previous clinical studies indicate that metabolic co-morbidities affect the clinical presentation of malaria. In Ghana and Nigeria, asymptomatic malaria parasitemia was more prevalent in persons with diabetes compared to those without diabetes [12, 13]. In Sweden, diabetes, obesity, and metabolic syndrome were identified as risk factors for severe malaria in travellers and migrants from sub-Saharan Africa diagnosed with *P. falciparum* [14]. However, it is unknown whether these comorbidities also affect malaria severity in a malaria-endemic area, where adults have developed some degree of immunity towards severe malaria.

Cameroon is endemic for malaria, and the prevalence of metabolic diseases in the country is on the rise. An estimated 774,200 adults were living with diabetes in 2024 [3]. A systematic review from Cameroon found that 26% and 15% of the adults have overweight and obesity, respectively [15]. In 2023, Cameroon accounted for 3.0% of cases and 1.9% of malaria deaths globally [5]. This study aimed to determine the prevalence of metabolic co-morbidities among adults with malaria and to investigate whether diabetes, obesity, and metabolic syndrome are associated with severe malaria in adults at the two most visited hospitals in the West region of Cameroon.

## Methods

### Study design and setting

We conducted a hospital-based cross-sectional study in the Dschang Regional Hospital Annex and the Bafoussam Regional Hospital in the West Region of Cameroon. The Dschang Regional Hospital Annex, with 170 beds, is a reference for health facilities in the Menoua sub-division and also serves a large student population of the University of Dschang. Bafoussam Regional Hospital, with 250 beds, is the main reference health facility of the West Region. Both are equipped with an emergency department and a laboratory with a parasitology unit.

### Study population

Patients aged ≥ 21 years diagnosed with malaria were identified in the emergency units, outpatient departments, wards, and maternity wards. All participants provided informed oral and written consent. Inclusion occurred in Dschang from June 2022 to August 2023 and Bafoussam from February 2023 to August 2023.

### Sample size

Epi Info™ 7.2.2.6 was used to estimate the sample size. Using the prevalence of diabetes in the general population in Cameroon; 5.2% [3] and assuming that 15.6% of severe malaria cases will have diabetes (three times the general prevalence, based on the findings from patients diagnosed with malaria in Sweden [14]), a minimum of 272 malaria cases was required to demonstrate 80% statistical power using a two-sided test with 95% confidence interval. The Swedish study was used to guide sample size assessment, since it was the only relevant study we found with estimates of comorbidities in severe and non-severe malaria patients from hospital settings. In addition, the majority of the participants in this study were from malaria-endemic settings in sub-Saharan Africa.

### Clinical assessment

Sociodemographic and health data, including information on education, occupation, medical conditions and recent malaria treatment, were collected with a questionnaire (Additional file 1) that we developed using Epi Info™ (version 7.2.2.6). Medical records were reviewed to capture concurrent diagnoses. Symptoms at presentation were documented, as were vital signs at first clinical assessment, including body temperature, blood pressure, respiratory rate, oxygen saturation, and Glasgow Coma Scale (GCS). Weight, height, and waist circumference were measured in all patients except pregnant women.

### Laboratory assessment

Malaria diagnosis was performed with rapid diagnostic testing (mRDT) (SD Bioline™ Pf/Pan) or microscopy using standard Giemsa staining. Both mRDT and microscopy are rarely requested for the same patient during routine health care services. When possible, we ordered additional malaria microscopy to determine the parasitemia in patients who initially tested positive with mRDT. To assess malaria severity, complete blood count, lipid profile, bilirubin, and creatinine were analysed for all patients in the hospital laboratories. Lactate was measured on venous blood using Lactate Pro® (Arkray factory, Inc.). Glycated haemoglobin (HbA1c) was determined using the Haemocue® HbA1c 501 point-of-care device and capillary blood glucose with a glucometer (MyLife Pura®). Sickle cell trait was analysed with haemoglobin electrophoresis, and data on the blood group were either available through medical records or tested in the hospital laboratory. HIV rapid diagnostic tests were performed following the national algorithm.

### Definition of severe malaria

Severe malaria was defined according to the 2022 WHO criteria (Additional file 2) [16]. For a stricter severity assessment, we used WHO criteria without prostration.

### Definition of comorbidities

The primary exposure variables were diabetes, obesity, and metabolic syndrome. A documented history of diabetes and IDF criteria were used to define diabetes [3]. Since HbA1c could be affected by acute malaria, patients with HbA1c ≥ 6.5% and normal blood glucose were contacted after discharge for confirmatory diabetes testing using fasting plasma glucose.

Body mass index (BMI) was calculated using weight [17]/height (m)^2^ [17] and categorised according to the WHO BMI classification for adults [18]. Obesity was defined as a BMI ≥ 30 kg/m^2^. BMI was not evaluated for pregnant participants.

Metabolic syndrome was determined following the criteria recommended by the IDF [19]:

- central obesity (waist circumference ≥ 94 cm in males and ≥ 80 cm in females; and/or BMI ≥ 30 kg/m^2^),
- plus, any two of:
  i) raised fasting blood glucose (FBG) (≥100mg/dL) or known diagnosis of diabetes,
  ii) elevated blood pressure (systolic blood pressure ≥130mmHg, diastolic ≥85mmHg, or being treated for hypertension)
  iii) reduced high-density lipoprotein (HDL) cholesterol (<40mg/dL in males and <50mg/dL in females),
    and
  iv) raised triglycerides (≥150mg/dL)

### Data management

A structured questionnaire was designed using Epi Info™ software to collect sociodemographic and clinical data. Subsequently, the data was entered into Sensivo® software (Sensivo Inc.) after pseudonymisation.

### Statistical analysis

The primary outcome was severe malaria. Statistical analyses were performed using STATA® version 16.1 software. Categorical data were compared using Chi-square or Fisher´s exact tests. Means and medians were compared using the t-test and the Mann–Whitney U test. Univariable and multivariable logistic regression were used to assess if diabetes, obesity, or metabolic syndrome were associated with severe malaria (with and without the criteria of prostration), expressed as odds ratios (OR) and adjusted odds ratios (aOR) with 95% confidence interval (CI). Missing data were reported in tables and excluded from models. Age group and sex were included as possible confounders in all multivariable analyses, as was HIV infection, given its known association with severe malaria [20, 21]. Additional patient characteristics associated with protection or risk for severe malaria in univariable analysis (p < 0.05) were included in the multivariable model to further adjust for confounders. The best model was selected based on the Hosmer–Lemeshow goodness-of-fit test. Stratified analyses were performed to assess the potential interactions between diabetes and obesity and diabetes and age. We also evaluated whether diabetes and obesity were associated with any specific criteria of severe malaria. P-values < 0.05 were considered statistically significant.

### Patient and public involvement

Considering the nature of this study, which included acutely ill patients with ongoing symptoms of malaria, patients could not be involved in the study design or preparation of the study protocol. Before the implementation of the protocol, the hospital management was consulted to give input that would fine-tune the patient inclusion procedure in the hospital. The research and its objectives were presented to all hospital units managing malaria to raise awareness of the ongoing study and obtain suggestions on the data collection process.

## Results

### Patient characteristics

**Fig. 1:**
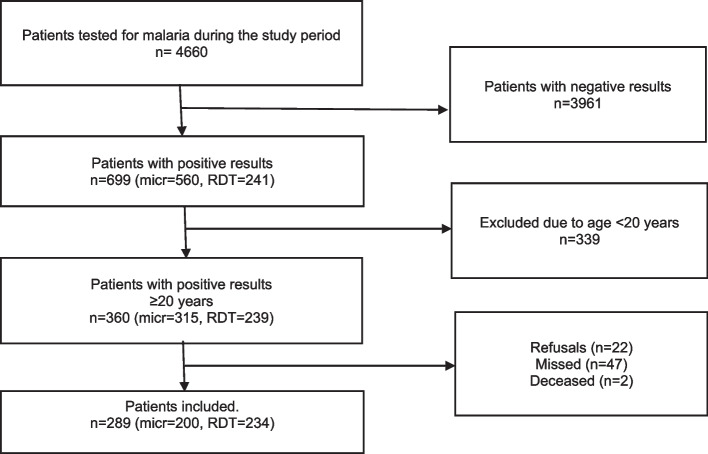
Flow chart of patient inclusion. Legend: Micr = malaria microscopy; RDT = rapid diagnostic testing for malaria. Missed patients were diagnosed with malaria, given treatment, and sent home (n = 47) or died (malaria-unrelated) before the investigators could include them (n = 2). A positive result refers to positive malaria RDT and/or positive malaria microscopy. For 145 of the 289, microscopy and RDT were performed for the same patients. All microscopy-positive cases were assessed as *Plasmodium falciparum*

Two hundred and eighty-nine (289, 80.3%) of the 360 adults diagnosed with malaria during the study period consented to participate (Fig. 1).

The mean age was 40.6 (± 19.3) years, with the predominant age groups being 20–29 years (124/289; 42.9%) and ≥ 60 years (69/289; 23.9%). Females comprised 63% (182/289) of the study population, and 17.6% (32/182) were pregnant. The age and sex composition of the individuals not included in the study were similar (Additional file 3).

Approximately 30% (86/289) of patients were students, with the majority (74/86; 86%) in Dschang. Other common occupations were farming (69/289; 23.9%) and business activities (67/289, 23.2%). Table 1 provides detailed patient characteristics. Biochemical characteristics of the patients are presented in Additional file 4.

**Table 1:** Characteristics of the study population according to malaria severity

Characteristic	Total (*N* = 289)	Non-severe malaria (N = 169)	Severe malaria (N = 120)	*p*-value	Crude OR (95%CI)
Age (years); mean, SD	40.6 ± 19.3	38.0 ± 17.6	44.2 ± 21.2	0.007	
Age (categorised)					
20–29	124 (42.9)	77 (45.5)	47 (39.2)		
30–39	48 (16.6)	30 (17.8)	18 (15.0)	0.962	1.02 (0.51–2.03)
40–49	24 (8.3)	18 (10.7)	6 (5.0)	0.282	0.58 (0.22–1.57)
50–59	24 (8.3)	17 (10.1)	7 (5.8)	0.496	0.72 (0.27–1.87)
≥ 60	69 (23.9)	27 (16.0)	42 (35.0)	0.002	2.55 (1.39–4.66)
Sex					
Male	107 (37.0)	69 (40.8)	38 (31.7)		
Female	182 (63.0)	100 (59.2)	82 (68.3)	0.089	1.53 (0.94–2.51)
Education					
None	17 (5.9)	4 (2.4)	13 (10.8)		
Primary	63 (21.8)	32 (18.9)	31 (25.8)	0.060	0.31 (0.09–1.05)
Secondary	42 (14.5)	19 (11.2)	23 (19.2)	0.153	0.39 (0.11–1.41)
High school	43 (14.8)	24 (14.2)	19 (15.8)	0.035	0.25 (0.07–0.91)
Higher education	124 (42.9)	90 (54.2)	34 (28.3)	0.000	0.12 (0.04–0.38)
Occupation					
Student	86 (29.8)	60 (35.5)	26 (21.7)		
Health worker	20 (6.9)	16 (9.5)	4 (3.3)	0.426	0.62 (0.19–2.03)
Farming	69 (23.9)	34 (20.1)	35 (29.2)	0.008	2.45 (1.26–4.74)
Business	67 (23.2)	32 (18.9)	35 (29.2)	0.005	2.61 (1.34–5.08)
Others	47 (16.2)	27 (16.0)	20 (16.6)	0.155	1.71 (0.81–3.57)
Comorbidities reported by patients or recorded in medical files					
Diabetes	15 (5.2)	4 (2.4)	11 (9.2)	0.016	4.23 (1.31–13.7)
Hypertension	30 (10.4)	17 (10.1)	12 (10.0)	0.255	1.35 (0.80–2.28)
Liver disease^1^	4 (1.4)	3 (1.8)	1 (0.8)	0.912	1.06 (0.40–2.77)
Asthma	2 (0.7)	2 (1.2)	0		
Cardiac disease^2^	4 (1.4)	3 (1.8)	1 (0.8)	0.350	1.78 (0.53–5.93)
Cancer^3^	4 (1.4)	0	4 (3.3)	0.581	1.21 (0.62–2.35)
Positive HIV test at inclusion^4^	10 (3.6)	5 (3.1)	5 (4.4)	0.568	1.45 (0.41–5.11)
Pregnancy	32 (17.6)	21 (21.0)	11 (13.4)	0.641	0.87 (0.49–1.54)
Hemoglobin traits					
Sickle-cell traits					
AA	243 (84.1)	142 (84.0)	101 (84.2)		
AS	27 (9.3)	18 (10.7)	9 (7.5)	0.383	0.69 (0.30–1.59)
SS	3 (1.1)	1 (0.6)	2 (1.7)	0.411	2.75 (0.25–30)
Missing	16 (5.5)	8 (4.7)	8 (6.6)		
Blood group^5^					
A	68 (22.8)	41 (24.3)	26 (21.7)		
B	56 (19.4)	36 (21.3)	19 (14.7)	0.631	0.72 (0.03–8.45)
AB	13 (4.5)	6 (3.6)	7 (5.8)	1.00	1.53 (0.05–19)
O	129 (44.6)	73 (43.2)	56 (46.7)	0.866	0.78 (0.05–12)
Missing	23 (7.9)	11 (6.5)	12 (10.0)		
Health behavioural factors					
Patient delay in consultation, days from symptom onset					
0–1	36 (13.6)	21 (12.6)	15 (12.5)		
2–3	113 (42.6)	67 (40.4)	46 (38.3)	0.919	0.96 (0.45–2.06)
≥ 4	116 (43.7)	67 (39.6)	49 (40.8)	0.889	1.06 (0.49–2.25)
Missing	24 (8.3)	14 (8.3)	10 (8.3)		
Malaria home treatment	82 (28.4)	49 (29.0)	33 (27.5)	0.742	0.92 (0.54–1.54)
Missing	1 (0.3)		1 (0.8)		

Legend: Data is presented as numbers and percentages (%) if not otherwise stated.

SD = Standard deviation

^1^cholecystitis, chronic hepatitis B and hepatitis C, steatohepatitis

^2^ heart failure, left ventricular hypertrophy, and cardiomyopathy

^3^uterus, breast, multiple myeloma, prostate; ^4^Nine out of these 10 cases were previously known; ^5^A + = 66, A- = 1, A (unconfirmed Rhesus) = 1, B + = 55, B- = 1, AB + = 12, AB- = 1, O + = 127, O- = 2

### Comorbidities

**Fig. 2:**
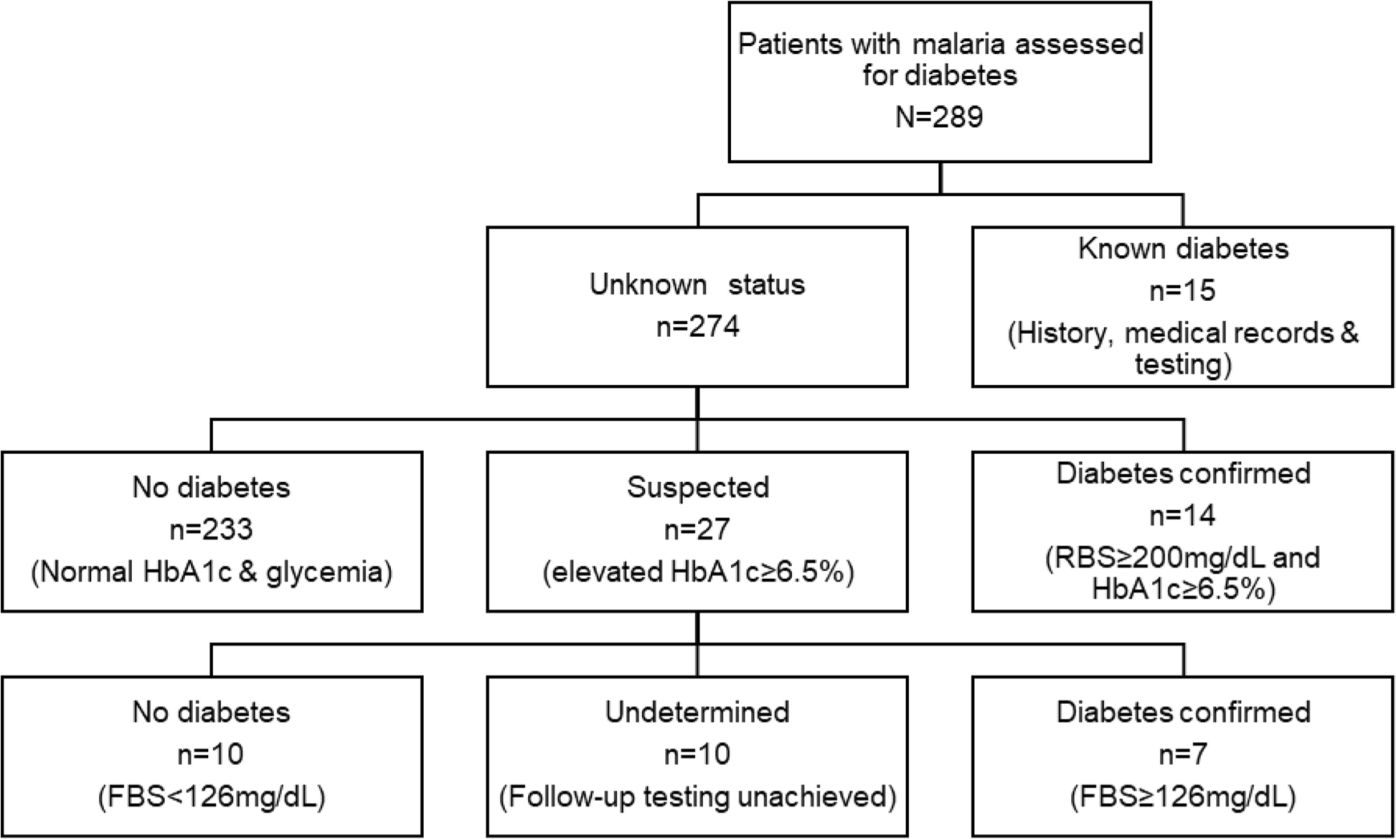
Flow chart of the identification and confirmation of diabetes. Legend: Abbreviation: RBS; random blood sugar, FPG; Fasting plasma glucose. *Four persons were not reachable by phone to call back for follow-up testing; one patient did not show up for the follow-up testing, while close family members declared 5 patients deceased since after discharge from the hospital

In total, 36 of 289 (12.5%) patients had diabetes, 15 (41.7%) of which were previously known and on antidiabetic treatment, based on patient history and medical records. Fourteen patients had abnormal random blood sugar and elevated HbA1c at admission, which fulfilled the definition of diabetes. An additional seven patients with elevated HbA1c were confirmed to have diabetes in follow-up testing with FBS after discharge (Fig. 2).

Twenty-six (21.7%) of the 120 severe malaria cases had diabetes, compared to 10 (6.0%) of 169 non-severe malaria cases (*p* < 0.001). BMI ≥ 30 kg/m^2^ was found in 57 of 289 (19.7%) patients, 23/120 (19.2%) vs 34/169 (20.1%) of severe and non-severe malaria cases, respectively (*p* = 0.848). Seventy-five of 289 (26.0%) patients had abnormal waist circumference, 39/120 (32.5%) severe and 36/169 (21.3%) non-severe malaria cases, respectively (*p* = 0.199). Central obesity (abnormal waist circumference or BMI ≥ 30 kg/m^2^) was found in 100 of 289 (34.6%) patients assessed, 47/120 (39.2%) vs 51/169 (30.2%) of severe and non-severe malaria cases, respectively (*p* = 0.136). Among the other elements that define metabolic syndrome, except for raised FBG, reduced HDL cholesterol (*p* = 0.021), and raised triglycerides (*p* = 0.003) were more prevalent in patients with severe malaria. The overall prevalence of metabolic syndrome was 68/289 (23.5%), found in 39/120 (32.5%) vs 29/169 (17.2%) of severe and non-severe malaria cases, respectively (*p* = 0.001) (Table 2).

**Table 2:** Clinical and laboratory profiles of patients at admission, including assessment of metabolic comorbidities according to malaria severity

Characteristic	Total (N = 289)	Non-severe malaria (N = 169)	Severe malaria (N = 120)	*p*-value	Crude OR (95% CI)
Diabetes					
Diabetes (new and known)	36 (12.5)	10 (5.9)	26 (21.7)	< 0.001	4.31 (1.99–9.34)
New diagnosed diabetes	21 (7.3)	6 (3.6)	15 (12.5)	0.007	3.81 (1.43–10)
Diabetes diagnosed at admission^1^	14 (4.8)	6 (3.6)	8 (6.7)	0.153	2.3 (0.73–7.21)
Diabetes confirmed at follow-up^2^	7 (46.7)	2 (25.0)	5 (71.4)	0.085	7.50 (0.76–74)
Diabetes (undetermined)	10 (3.5)	4 (2.4)	6 (5.0)	0.238	2.17 (0.60–7.87)
Metabolic syndrome criteria (n = 264)					
Body-mass index (BMI)					
Mean BMI (kg/m^2^), SD	26.7, 6	26.6, 6	26.6, 5	0.934	
Underweight (< 18 kg/m^2^)	8 (2.8)	5	3 (2.5)		
Normal (18–24.9 kg/m^2^)	105 (36.3)	62 (37.4)	43 (35.8)	0.848	1.15 (0.26–5.09)
Overweight (25–29.9 kg/m^2^)	80 (27.7)	45 (27.1)	34 (28.3)	0.763	1.26 (0.28–5.64)
Obese (≥ 30 kg/m^2^)	57 (19.7)	33 (19.9)	23 (19.2)	0.848	1.16 (0.25–5.35)
Missing	39 (13.5)	21 (12.6)	17 (14.2)		
Waist circumference^3^					
Normal	83 (28.7)	52 (31.3)	31 (25.8)		
Abnormal	75 (26.0)	36 (21.3)	39 (32.5)	0.199	1.87 (0.99–3.53)
Missing	131 (45.3)	81 (47.9)	50 (41.7)		
Hypertension^4^,	92 (32.2)	57 (34.3)	35 (29.2)	0.744	1.10 (0.62–1.96)
Reduced HDL cholesterol^5^	191 (70.2)	106 (65.8)	85 (76.6)	0.057	1.70 (0.98–2.93)
Raised Triglycerides^6^	115 (43.1)	54 (34.4)	61 (55.5)	0.001	2.37 (1.44–3.91)
Raised fasting blood glucose^7^	87 (36.2)	39 (28.1)	48 (47.5)	0.002	2.32 (1.36–3.98)
Metabolic syndrome	68 (25.8)	27 (17.6)	39 (35.8)	0.001	2.6 (1.47–4.60)

Data is presented as numbers and percentages (%) if not otherwise stated

^1^ ≥ 6.5%, RBS > 200 mg/dL)

^2^ These patients on admission had HbA1c ≥ 6.5 but RBS < 200 mg/dL and were tested for fasting plasma glucose (FPG ≥ 126 mg/dL) weeks after discharge from the hospital

^3^ Abnormal implies waist circumference ≥ 94 cm in men and ≥ 80 cm in women

^4^ known or SBP ≥ 130 mmHg on admission

^5^ ≤ 40 mg/dL (men), ≤ 50 mg/dL (women), ^6^ > 150 mg/dL, ^7^ ≥ 100 mg/dL

The overall HIV prevalence was 10/289 (3.5%), 9/10 (90%) of them on antiretroviral treatment, and 5/120 (4.2%) in severe compared to 5/169 (3.0%) in non-severe malaria cases (p = 0.568) (Table 1).

### Presentations and factors associated with severe malaria

Severe malaria, based on all WHO criteria, was found in 120/289 (41.5%) patients. Most of the severe malaria cases (87/120, 72.5%) showed signs of prostration; other common criteria were lactic acidosis (37.1%), impaired consciousness (14.4%), hyperbilirubinemia without hyperparasitemia (8.0%) and severe anaemia (15.3%). Patients with obesity alone (by BMI) and patients with both obesity and diabetes had a higher prevalence of pulmonary oedema (7/44; 15.9%, p = 0.005, and 4/13; 30.8%, p = 0.003, respectively). Severe anaemia and acidosis were more common among patients with diabetes than in patients without (Table 3).

**Table 3:** WHO severe malaria criteria and other severity signs, stratified by comorbidity group (diabetes, obesity^5^, and both) in patients with severe malaria

Severity criteria	Total *N* = 289 n	Diabetes, all *N* = 36		Diabetes**,** no obesity *N* = 18		Obesity, no diabetes (by BMI) *N* = 44		Diabetes and Obesity *N* = 13	
		n (%)	*p*-value	n (%)	*p*-value	n (%)	*p*-value	n (%)	*p*-value
WHO criteria	120	26 (72.2)	< 0.001	12 (66.7)	0.025	13 (30.2)	0.080	10 (76.9)	0.018
WHO criteria minus prostration	76	19 (52.8)	< 0.001	8 (44.4)	0.071	12 (27.3)	0.873	7 (53.9)	0.021
Impaired consciousness	18	4 (11.1)	0.369	1 (5.9)	0.339	2 (4.6)	0.787	3 (16.7)	0.603
Multiple convulsions	8	1 (2.7)	0.991	0	1.00	0	0.613	1	1.00
Severe anaemia^1^	18	5 (13.9)	0.034	4 (22.2)	0.016	0	0.085	1	0.888
Acidosis	23	7 (41.2)	0.003	4 (22.2)	0.175	3 (13.6)	1.00	3 (50.0)	0.059
Pulmonary edema	18	4 (12.5)	0.190	0	0.610	7 (15.9)	0.005	4 (30.8)	0.003
Shock	7	0	0.301	0	1.00	2 (4.6)	0.292	0	1.00
Hyperparasitemia^2^	2	0	1.00	0	1.00	0	1.00	0	1.00
Bilirubin ≥ 3 mg/dL^3^	14	2 (6.5)	0.668	2 (13.3)	0.179	2 (4.8)	1.00	0	1.00
Creatinine > 5 mg/dL	1	0	1.00	0	1.00	0	1.00	0	1.00
Kidney injury^4^ Stages 2 and 3	13	3 (8.3)	2.11	2 (11.1)	0.190	1 (2.3)	0.699	1 (5.6)	0.574

^1^ at < 10,000 trophozoites^/^µL

^2^100,000 trophozoites/µL

^3^with/without 100,000 trophozoites/uL

^4^according to the KDIGO criteria

^5^excludes pregnant women with diabetes because obesity was not assessed in pregnant women

**Fig. 3:**
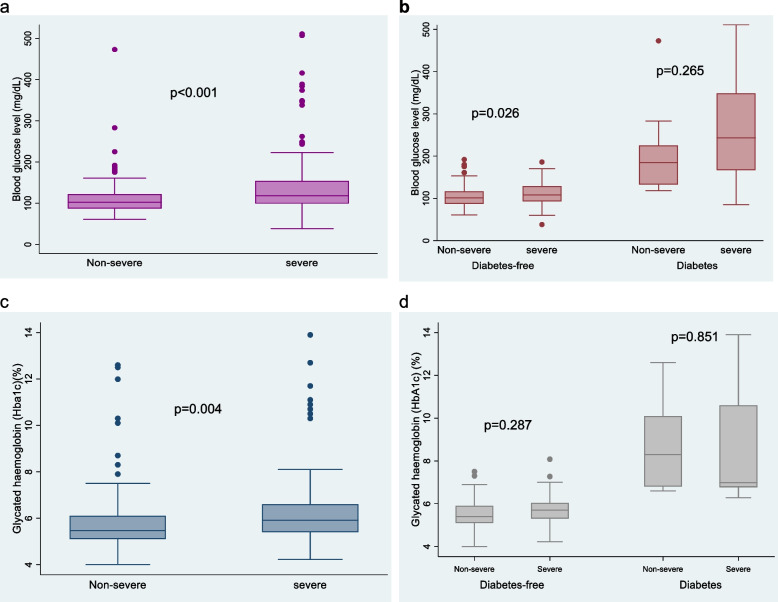
On-admission blood glucose level and HbA1c according to malaria severity and diabetes status. Legend: P-value calculated with the t-test. P-value calculated with the t-test

In univariable analysis, age ≥ 60 years (OR = 2.55; 95% CI, 1.39–4.66), farming (OR = 2.45; 95% CI, 1.26–4.74) and business works (OR = 2.61; 95% CI, 1.34–5.08) were associated with severe malaria, while high school or higher education was associated with reduced likelihood of severe malaria. Diabetes (OR = 4.51; 95% CI, 2.11–9.93) and MetS (OR = 2.60; 95% CI, 1.47–4.60) increased the odds of severe malaria, but not HIV (OR = 1.42; 95% CI, 0.40–5.01). We found no association between obesity (neither by BMI nor waist circumference) and severe malaria. Sex and blood group were not significantly associated with malaria severity, and no protective effect for sickle cell trait was observed. Diabetes and MetS remained associated with severe malaria after adjusting for age group, occupation, educational level, and HIV status, with an OR = 3.24 (95% CI, 1.38–7.62) and 3.18 (95% CI,1.60–6.37), respectively. Higher mean blood glucose levels (p < 0.001) and higher proportions of HbA1c (*p* = 0.004) were observed in severe compared with non-severe malaria cases (Fig. 3).

When using restricted malaria severity criteria (without prostration), 76/289 (26.3%) patients fulfilled the definition. The proportion of diabetes was still significantly higher among severe (19/76, 25%) compared to non-severe cases (17/213, 7.9%) (*p* < 0.001). Age, educational level, and occupation were associated with restricted severe malaria, similar to the unrestricted definition. In univariate analysis, diabetes and MetS were still significantly associated with severe malaria without prostration. Interestingly, central obesity was associated with the stricter definition of severe malaria (OR = 1.80; 95% CI,1.05–3.08), while obesity by BMI alone was not. Diabetes, central obesity, and MetS remained associated with restricted severe criteria also after adjusting for age group, occupation, educational level and HIV status (Table 4).

**Table 4:** Metabolic comorbidities and association with severe malaria according to WHO and restricted criteria

Variable	Malaria severity according to all WHO criteria				Malaria severity using restricted criteria^9^			
	Non-severe malaria (*N* = 169), n (%)	Severe malaria (*N* = 120) n (%)	Crude OR (95%CI)	Adjusted OR (95%CI)	Non-severe malaria (*N* = 213), n (%)	Severe malaria (*N* = 76) n (%)	Crude OR (95%CI)	Adjusted OR (95%CI)
Confirmed Diabetes	10 (5.9)	26 (21.7)	4.58 (2.11–9.93)	3.24 (1.38–7.62)^1^	17 (8.0)	19 (25.0)	4.01 (1.95–8.25)	2.29 (1.02–5.15)^3^
Metabolic syndrome	28 (18.5)	37 (35.9)	2.60 (1.47–4.60)	3.18 (1.60–6.37)^2^	41 (20.9)	27 (35.5)	2.49 (1.37–4.52)	2.58 (1.26–5.31)^4^
Central obesity^10^	50 (30.3)	45 (39.5)	1.45 (0.89–2.38)	1.35 (0.76–2.44)^5^	66 (31.0)	34 (44.7)	1.80 (1.05–3.08)	2.52 (1.24–5.11)^6^
BMI ≥ 30 kg/m^2^	33 (22.9)	23 (23.5)	0.98 (0.54–1.80)	1.70 (0.91–3.17)^7^	38 (20.2)	19 (30.2)	1.70 (0.89–3.25)	1.46 (0.69–3.09)^8^

^1,2^adjusted for age group, education, occupation, and HIV status

^3,4,5,6,7,8^adjusted for age group, education, occupation, and HIV status

^9^without prostration

^10^central obesity is defined abnormal waist circumference or BMI ≥ 30 kg/m^2^

In stratified analysis by BMI (≥ 30 or < 30 kg/m^2^), we found an OR = 3.33 (95% CI, 1.19–9.32) for severe malaria (using all criteria) in persons with diabetes only and OR = 7.69 (95% CI, 1.81–32) in persons with both diabetes and obesity, indicating that obesity could modify the association with severity. When stratifying for age group, we observed that diabetes was associated with six times increased odds for severe malaria in patients ≥ 60 years (OR = 6.27; 95% CI, 1.61–24), but the association did not reach significance for the age group < 60 years (OR = 2.28; 95% CI, 0.79- 6.57) (Table 5), thus possibly also age could modify the ´ association between diabetes and severe malaria.

**Table 5:** Association of diabetes with severe malaria, stratified by obesity and age group

Severe malaria definition	Diabetes, no obesity *N* = 18		Diabetes and obesity *N* = 13		Diabetes, age < 60 *N* = 15		Diabetes, age ≥ 60 *N* = 21	
	n (%)	OR 95% CI	n (%)	OR (95%CI)	n (%)	OR 95% CI	n (%)	OR (95%CI)
All WHO criteria	12(66.7)	3.33(1.19–9.32)	10(76.9)	7.69(1.81–32)	8(53.3)	2.29(0.79–6.57)	18(85.7)	6.27(1.62–24.3)
Restricted criteria	8(44.4)	3.27(1.20–8.93)	7(53.9)	3.01(0.84–10)	4(26.7)	1.64(0.49–5.43)	15(71.4)	4.12(1.34–12.6)

## Discussion

Malaria, diabetes and metabolic syndrome are associated with significant morbidity and mortality. This study aimed to determine whether diabetes and other metabolic comorbidities could affect the severity of malaria in a malaria-endemic setting. Among 289 adult malaria patients diagnosed at two hospitals in Western Cameroon and systematically assessed for diabetes and metabolic syndrome, we identified an overall high prevalence of diabetes, of which 58% were previously undiagnosed. Furthermore, we found that the proportion of diabetes among severe malaria cases was almost three times higher than in non-severe cases. After adjusting for possible confounders, we showed that diabetes and metabolic syndrome were associated with severe malaria. We also found a high prevalence of obesity in the study population but no association with severe malaria when obesity was assessed by BMI only. Based on these findings, diabetes and metabolic syndrome could affect the disease presentation of malaria in semi-immune adults living in malaria-endemic countries.

Clinical studies evaluating the effect of NCDs on malaria in sub-Saharan Africa are rare. The few investigations carried out in endemic settings concern the potential risk of asymptomatic malaria infections in patients with diabetes. An earlier study from Ghana demonstrated a higher proportion of malaria among persons with diabetes compared to controls [12]. A cross-sectional health screening campaign in South Western Nigeria also found diabetes associated with *Plasmodium falciparum* infection [13], and a study in Lagos found persons with diabetes to have a high prevalence of asymptomatic malaria, but there was no comparison group [22]. Clinical evaluation of malaria symptoms and severity was not performed in these studies.

To our knowledge, this is the first sub-Saharan African study to assess the role of diabetes and metabolic comorbidities in the disease severity of malaria. The association of diabetes, obesity and metabolic syndrome with a several-fold increase in the risk of severe malaria has been reported in Sweden in non-immune travelers and migrants from malaria-endemic countries [15]. In contrast, a recent survey of imported malaria in Germany, with 61% of patients with endemic origin, demonstrated an association between hypertension and severe malaria but did not find any with diabetes or obesity. However, the prevalence of diabetes was low (3.0%; 16/536), and the survey lacked a systematic assessment of diabetes [30].

Obesity was assessed using BMI and waist circumference, which are strong cardio-metabolic risk predictors in sub-Saharan Africa [23]. Obesity by BMI alone was not associated with severe malaria, contrasting to the study from Sweden [14]. However, levels of adiposity and health risks related to BMI may differ depending on ethnicity [24]. Generally, waist circumference better predicts insulin resistance and the development of type 2 diabetes, but the etiological relationships between type 2 diabetes and central obesity may vary in different populations [23]. When abnormal waist circumference and/or BMI ≥ 30 kg/m^2^ were used to define central obesity, it was found to be associated with severe malaria using more restricted severity criteria. In addition, the stratified analysis indicated that patients with both type 2 diabetes and obesity have the highest risk of severe manifestations.

Even though individuals from malaria-endemic areas develop immunity to severe disease with age and repeated exposure, we observed an increased proportion of severe malaria in individuals aged ≥ 60. In non-endemic settings, old age is one of the most important risk factors for severe malaria [14, 21, 25]. However, how senior ageing affects naturally acquired immunity to malaria has not been well studied. Interestingly, the age effect seemed to disappear after adjustment for diabetes. However, stratified analysis demonstrated that among individuals with diabetes, old age was still associated with severe malaria. Diabetes is associated with low-grade inflammation and altered immune responses [26]. Possibly, long-term diabetes could affect acquired immunity to malaria in older individuals, or older individuals with diabetes have more severe disease due to poor glycemic control and diabetes-related vascular complications.

Severe malaria pathogenesis involves rosetting, the formation of aggregated, uninfected RBCs around infected RBCs [26, 27]. In diabetes, RBCs have also been shown to reversibly stack into rouleaux, which limit blood circulation in narrow capillaries [27]. An in vitro study demonstrated that parasite rosetting was enhanced with *P. falciparum* parasites cultured ex vivo in the blood of diabetic (non-infected) patients compared to non-diabetic controls [28]. Possibly, altered red blood cell properties in patients with diabetes could also contribute to the more severe malaria disease presentations observed in these patients.

Other risk factors systematically investigated were HIV status, blood group, and sickle-cell trait. HIV has been associated with severe malaria presentation in untreated HIV patients with low CD4 count [14, 20, 29]. In our study population, no association between HIV infection and malaria severity was observed, but almost all HIV patients were previously diagnosed and on antiviral treatment. Earlier studies have shown the protective effects of sickle cell traits [30] and Blood Group O [31] on severe malaria. Although the prevalence of severe malaria was slightly lower among HbAS patients, no association with severity was observed for HbAS or blood group.

There are some limitations to this study. Uncomplicated malaria cases might have sought care in other facilities outside the study sites. Furthermore, patients seeking care in the hospitals could have more comorbidities, potentially introducing selection bias. Secondly, the reliability of microscopy for diagnosing malaria in this setting is variable, and positive mRDTs can reflect previously treated malaria. To limit the uncertainty, both microscopy and RDT were used. However, approximately half of the cases were diagnosed with just one method. The difficulty in obtaining suitable malaria slides and variability in mRDT supply reflects the restricted diagnostic setting of most healthcare facilities in Cameroon. Future studies investigating the pathogenesis of severe malaria in patients with metabolic comorbidities will need more reliable diagnostics and possibly additional methods to distinguish severe malaria from other severe infections with concurrent parasitaemia, such as measurement of plasma PfHRP2 concentrations; this would, however, require another standard of health care facilities and laboratory equipment. Finally, malaria could affect the concentration of HbA1c and glucose during the acute episode. This was addressed by follow-up testing after discharge. Meanwhile, history and medical records also found a higher proportion of diabetes among severe malaria cases.

Mindful of these limitations, our findings uncover a double burden of highly prevalent NCDs and clinical malaria at the hospital level in an endemic area. In addition, patients were systematically assessed for metabolic comorbidities according to standardized international guidelines and thoroughly evaluated for clinical and laboratory signs of severe malaria and other traits and comorbidities that could affect malaria severity.

## Conclusion

The findings bear important clinical implications in sub-Saharan Africa, considering the high burden of malaria and the rising prevalence of diabetes and other metabolic comorbidities. The study adds evidence to previous observations that malaria severity in adults could be affected by diabetes and metabolic risk factors, also in individuals with partial immunity. The high prevalence of diabetes among patients hospitalized for malaria, of which a majority were previously undiagnosed, indicates that testing for diabetes, simply by measuring blood sugar at discharge, should be considered in adults admitted with malaria, particularly those with severe disease presentations. Studies from other malaria-endemic countries are warranted to confirm our results.

## Supplementary Information

Additional file 1: Diabetes and metabolic syndrome in adults with malaria –Questionnaire.

Additional file 2: WHO 2022 severity criteria for defining severe malaria.

Additional file 3: Comparison of sociodemographicof patients included and those not included in the study.

Additional file 4: Detailed biochemistry assessment of patients with malaria, according to WHO severe malaria criteria.

## Data Availability

Due to concerns about data privacy, the datasets produced and/or analysed for the present study are not currently openly available. Until the data is deposited in a repository, reasonable requests to access that data should be sent to the corresponding author.

## Abbreviations

adjOR: Adjusted odds ratio
BMI: Body mass index
CI: Confidence interval
CD4: Cluster of differentiation 4
FBG: Fasting Blood Glucose
GCS: Glasgow Coma Scale
HbA1c: Glycated haemoglobin
HbAS: Hemoglobin AS
HDL: High-density lipoprotein
HIV: Human Immunodeficiency Virus
IDF: The International Diabetes Federation (IDF)
KDIGO: Kidney Disease Improving Global Outcomes
Met-S: Metabolic Syndrome
mRDT: Malaria Rapid Diagnostic Testing
NCD: Non-communicable Disease
OR: Odds ratio
PfHRP2: *Plasmodium falciparum* Histidine-rich protein 2
RBC: Red Blood Cell
WHO: World Health Organization
