# Supplementary material for: Diabetes and metabolic syndrome in adults with malaria and associations with severe disease: results from two tertiary hospitals in Cameroon
Source: BMC Infect Dis. 2025 Aug 22;25:1060. doi: 10.1186/s12879-025-11389-1 (PMC12374314; doi:10.1186/s12879-025-11389-1)
Supplement: Supplementary file 1 — Additional file 1: Diabetes and metabolic syndrome in adults with malaria –Questionnaire. [file 12879_2025_11389_MOESM1_ESM.pdf]

Diabetes and metabolic syndrome in adults with malaria -Questionnaire.

Information about Data Collector

Initials: ______ Date of admission ______ Date of interview ______ Study site ______

Participant Identification and Sociodemogtraphic Information

Study ID: ______ Sample ID ______ Residence: ______ Age (yrs) ______

Sex
- ◯ male
- ◯ female

Educational level
- ◯ primary
- ◯ none
- ◯ high school/BAC/Probabtoire
- ◯ secondary/cap
- ◯ technical college/university

Occupation
- ◯ farming
- ◯ petit business
- ◯ health professional
- ◯ student
- ◯ others:
- ◯ others:

Medical Background

1. Do you have any of the following conditions?

i) Diabetes?
- ◯ yes
- ◯ no
- ◯ i dont know

ii) High blood pressure?
- ◯ yes
- ◯ no
- ◯ i dont know

2. For how long have you been diagnosed with these conditions?

i) Diabetes:
- ◯ ________(years)
- ◯ recent (less than 3 months)
- ◯ i dont know

ii) High blood pressuer:
- ◯ ________(years)
- ◯ recent (less than 3 months)
- ◯ i dont know

3. Are you on treatment for any of the following conditions? If Yes, which medication do you take?

i) Diabetes?
- ◯ yes (medication (s)____________)
- ◯ no
- ◯ i dont know

ii) High blood pressure?
- ◯ yes (medication (s)____________)
- ◯ no
- ◯ i dont know

4. Do you have other health complications such as;

i) Kidney disease?
- ◯ no
- ◯ yes (type____________)
- ◯ i dont know

ii) Liver problems?
- ◯ no
- ◯ yes (type____________)
- ◯ i dont know

iii) Lung problems?
- ◯ no
- ◯ yes (type____________)
- ◯ i dont know

v) Heart or other cardiovascular problems?
- ◯ no
- ◯ yes (type____________)
- ◯ i dont know

iv) HIV
- ◯ no
- ◯ i dont know
- ◯ yes

5. Have you ever smoked cigarettes?
- ◯ no
- ◯ yes

6. Do you currently smoke cigarettes?
- ◯ no
- ◯ yes

7. Are you pregnant?
- ◯ no
- ◯ i dont know
- ◯ yes

8. Was malaria treatment taken at home?
- ◯ no
- ◯ yes

9. Type of home treatment
- ◯ ________
- ◯ dont remember

doses ______

10. List all additional present medications ______

Clinical Assessment on Admission/Consultation Day

i. Symptoms experienced

For how long (days) have you been sick? ______

Symptom		
chills	◯ yes	◯ no
Fever	◯ yes	◯ no
Body weakness	◯ yes	◯ no
Headache	◯ yes	◯ no
Vomiting	◯ ye	◯ no
Diarrhoea	◯ yes	◯ no
Abdominal pains	◯ yes	◯ no
Cough	◯ yes	◯ no
Neurologic symptoms	◯ yes	◯ no
History of convulsions	◯ yes	◯ no
Dark urine	◯ yes	◯ no
low urine volumes	◯ yes	◯ no

ii. Clinical signs

Sign		
Prostration	◯ yes	◯ no
Jaundice	◯ yes	◯ no
Anemia	◯ yes	◯ no
Multiple convulsions	◯ yes	◯ no
Impaired consciousness	◯ yes	◯ no

Respiratory distress
○ yes ○ no

Lung crackles
○ yes ○ no

Shock
○ yes
○ no

Significant bleeding
○ yes ○ no

Haematuria
○ yes
○ no

Low/no urine output
○ yes ○ no

Vital parameters and the anthropometric measures

Glasgow Coma Scale=[__________]/15
○ eye opening=[____]/4 ○ motor responce=[____]/6
○ verbal response=[____]/5

Temperature (°C) [ ] Pulse rate (bpm) [ ]
Respiration (br/min) [ ] BP (mmHg) [ ]
Weight (kg) [ ] Height (cm) [ ]

Waist circumference (cm) [ ] BMI (kg/m2) [ ] Oxygen saturation [ ]

Laboratory Findings on Admission/Consultation Day (day 1)

#Malaria parasite (per uL) [ ]

Fasted measurements
○ blood glucose only ○ lipds only
○ both ○ none

Plasmodium species
○ P. falc ○ P. ova
○ P. mal ○ others

Malaria RDT
○ neg
○ pos

RDT Plasmodium species
○ PF
○ others

Date sample collected [ ] Blood glucose (mg/dL) [ ]

WBC count (x10^3) [ ] Hemoglobin (g/dL) [ ] Hematocrit (%) [ ] Platelet count (x10^6) [ ]

Total bilirubin (mg/dL) [ ] Serum creatinine (mg/dL) [ ] Total triglyceride (g/L) [ ] HDL (g/L) [ ]

HBA1c(%) [ ] HB electrophoresis result [ ]

Blood group
○ O | Rh___ ○ A | Rh___ ○ B | Rh___ ○ AB | Rh___

HIV status
○ neg ○ pos

Venous pH [ ]

Results of laboratory check for other (co-) infections;
○ Urinalysis: ____________ ○ Stool exam: ____________ ○ others
○ C-reactive protein: ____________ ○ Radiology result: ____________

Patient Management Decision

Malaria severity
○ uncomplicated
○ severe
○ not documented

Other diagnosed/suspected infections
○ 1. ____________
○ 2. None

Care location
○ sent home
○ admitted

Reason (s) for admission
○ 1.____________
○ 2.____________

Inpatient Follow-up

Fasting blood sugar
○ day 2 admission: ______
○ day 3 admission:

Hemoglobin (g/dL)
○ day 2: ______
○ day 3: ______

Malaria parasitemia (trop/uL)
○ day 2: ______
○ day 3: ______

ICU care?
○ no ○ yes

Name of first malaria drug [ ]

Route of first malaria drug
○ IV ○ IM ○ PO

Number of doses
○ IV=[___] ○ IM=[___]

Name of follow-up oral malaria drug [ ]

Was (were) any antibiotic (s) given?
○ no ○ yes

If yes, name of the antibiotic (s) [ ]

Other drugs given [ ]

Supportive treatments provided
○ oxygen
○ blood transfusion
○ fluids and others
○ no supportive tx

Days hospitalised
○ Ward=[___] ○ ICU=[___]

Date discharged [ ]

Complications during admission
○ renal disfunction ○ impaired consciousness ○ others: __________
○ severe anemia ○ diceased ○ no complication
○ bleeding ○ lung oedema and ARDS
