# Supplementary material for: Diabetes and metabolic syndrome in adults with malaria and associations with severe disease: results from two tertiary hospitals in Cameroon
Source: BMC Infect Dis. 2025 Aug 22;25:1060. doi: 10.1186/s12879-025-11389-1 (PMC12374314; doi:10.1186/s12879-025-11389-1)
Supplement: Supplementary file 2 — Additional file 2: WHO 2022 severity criteria for defining severe malaria. [file 12879_2025_11389_MOESM2_ESM.pdf]

Additional File 2: WHO 2022 severity criteria for defining severe malaria

Criteria	**Explanation[β]**
Impaired consciousness	Glasgow Coma Score <11
Prostration	Generalised weakness so that the person is unable to sit, stand, or walk unaided
Multiple convulsions	More than two episodes within 24 hours
Acidosis	Venous plasma lactate >= 5 mmol/L
Hypoglycaemia	Bood or plasma glucose < 2.2 mmol/L (< 40 mg/dL)
Severe malarial anemia	Haemoglobin <7g/dL and haematocrit <20%, with a parasite count > 10,000/µL
Renal impairment	Plasma or serum creatinine >265 µmol/L(3mg/dL)
Jaundice	Plasma or serum bilirubin >50µmol/L (3mg/dL) with a parasite count >100 000/µL
Pulmonary edema	Radiologically confirmed or oxygen saturation <92% on room air with a respiratory rate >30/min, often with chest in-drawing and crepitations on auscultation.
Significant bleeding	Including recurrent or prolonged bleeding from the nose, gums, or venepuncture sites; hematemesis or melena
Hyperparasitemia	*Plasmodium falciparum* parasitemia>10%
Shock	Decompensated (systolic blood pressure <80mmHg in adults) with evidence of impaired perfusion

[β]WHO Guidelines for malaria, 25 November 2022. Geneva: World Health Organization; 2022 (WHO/UCN/GMP/2022.01 Rev.3). License: CC BY-NC-SA 3.0 IGO
