# Supplementary material for: Diabetes and metabolic syndrome in adults with malaria and associations with severe disease: results from two tertiary hospitals in Cameroon
Source: BMC Infect Dis. 2025 Aug 22;25:1060. doi: 10.1186/s12879-025-11389-1 (PMC12374314; doi:10.1186/s12879-025-11389-1)
Supplement: Supplementary file 3 — Additional file 3: Comparison of sociodemographicof patients included and those not included in the study. [file 12879_2025_11389_MOESM3_ESM.pdf]

Additional File 3: Comparison of sociodemographic (age and sex) of patients included and those not included in the study

	Included (n=289)	Not included (n=73)	
Age group	n (%)	Total, n (%)	p-value[1]
20-29	124(42.9)	26 (35.6)	0.941
30-39	48(16.6)	18(24.7)	
40-49	24(8.3)	7(9.6)	
50-59	24(8.3)	9(12.3)	
≥60	69(23.9)	13 (17.8)	
Sex			
Male	107(37.0)	25(34.2)	0.660
Female	182(63.0)	48(65.8)	
Location			
Bafoussam	81(28.0)	28(38.4)	0.086
Dschang	208(72.0)	45(61.6)	

[1]p-value from analysis of the linear trend in proportions
