# Supplementary material for: Diabetes and metabolic syndrome in adults with malaria and associations with severe disease: results from two tertiary hospitals in Cameroon
Source: BMC Infect Dis. 2025 Aug 22;25:1060. doi: 10.1186/s12879-025-11389-1 (PMC12374314; doi:10.1186/s12879-025-11389-1)
Supplement: Supplementary file 4 — Additional file 4: Detailed biochemistry assessment of patients with malaria, according to WHO severe malaria criteria. [file 12879_2025_11389_MOESM4_ESM.pdf]

Additional File 4: Detailed biochemistry assessment of patients with malaria, according to WHO severe malaria criteria.

Characteristic	Total (N=289)	Non-severe malaria (N=169)	Severe malaria (N=120)	p-value
**Blood biochemical parameters**				
Lactate(mmol/L) (median, IQR)	4.2(2.8-5.6)	2.4(2.1-2.6)	6.2(3.4-9.0)	0.005[1]
Lactate ≥5mmol/L	23(16.8)	0	23(37.1)	<0.001
Total bilirubin (mg/dL median, IQR)	0.98(0.52-1.64)	0.91(0.49-1.57)	1.09(0.54-1.77)	0.472[1]
Tota bilirubin >3 mg/dL	14(5.2)	5(3.2)	9(8.0)	0.319
Blood glucose (g/dL) (mean, SD)	126, 67	110, 3.44	146, 8.20	<0.001[1]
Creatinine (median, IQR)	1.04(0.81-1.32)	1.02(0.8-1.31)	1.1(0.89-1.38)	0.450[1]
Creatinine >5mg/dL	1(0.4)	0	1(0.9)	
KDIGO assessment of Kidney injury				
Stage 1	16(5.7)	9(5.5)	7(6.0)	0.782
Stage 2	7(2.5)	3(1.8)	4(3.4)	0.378
Stage 3	6(2.1)	1(0.6)	5(4.3)	0.069
**Glycated hemoglobin (HBA1c)**				
Mean, SD	6.04, 1.49	5.82, 1.31	6.35, 1.66	0.005[2]
HBA1c categories				
4-5.6	126(48.5)	89(58.5)	37(34.3)	<0.001
5.7-6.4	74(28.5)	39(25.7)	35(32.4)	
≥6.5	60(23.1)	24(15.8)	36(33.3)	
**Haematological parameters**				
Leucocytes(10^3 /L) (median, IQR)	5300 (4330-6900)	5000 (2900-6550)	5700(4150-7600)	0.428[1]
Hemoglobin(g/dL) (median, IQR)	12.4(12.1-12.7)	12.8(12.5-13.1)	11.8 (11.3-12.3)	<0.001[1]
Hemoglobin <7g/dL	18(6.4)	0	18(15.3)	<0.001
Platelets (median, IQR)	132 (84-190)	143(93-200)	113(72-167)	0.602[1]
$<150*10^9$ /L	160(59.3)	82(53.6)	78(66.7)	0.041
$\geq150*10^9$ /L	110(40.7)	71(46.4)	39(33.3)	

[1] Wilcoxon rank-sum test; [2] t-test
